# Breast augmentation

**Published:** 2008-10

**Authors:** K. Ramachandran

**Affiliations:** Senior Consultant Cosmetic Surgeon, Apollo Hospitals, 21 Greames Lane, Chennai - 600 006, India

## INTRODUCTION

The female breast has been synonymous with femininity and hence a lot of focus has been given to the aesthetics of the organ. The ideal size and shape vary, depending upon the build of the individual and the cultural characteristics. Many a time, breast development does not take place adequately. As a result, women with smaller than normal breasts feel that they have a disproportionate figure and therefore seek correction through surgery. It is, therefore, important that the surgeon also takes into consideration the patient's desires, when planning an augmentation surgery. Breast augmentation can have significant positive influence on the body image.

## INDICATIONS

Many women seek breast enlargement in order to correct hypoplastic breasts. Those who have undergone significant post partum involution also opt for augmentation, for further improvement. These women have experienced the fullness and want the volume back. Some women opt for surgery for correcting asymmetry.

Since the introduction of silicone gel prosthesis in 1962, breast augmentation has become the most commonly performed operation in cosmetic surgery.

## HISTORY

Czerny attempted the first augmentation mammoplasty, in which he transferred a lipoma to the breast, in 1895.[1] Longacre[2] performed autogenous flap augmentation in 1950. There were many injectable materials that were being tried, since the 1950s. Uchida[3] reported the use of injectable silicone in 1961. The introduction of the silicone gel breast implant in 1962 by Cronin and Gerow[4] initiated the modern era of breast augmentation.

## EVOLUTION OF MAMMARY IMPLANT

### Silicone gel implants

The development of silicone progressed to meet the needs of the aircraft-engineering industry during World War II. Being soft and inert, it attracted interest from the medical field too. First generation implants (1962-1970) had thick shells, thick gel, and a Dacron patch in the posterior aspect. It had a tear drop shape. Second generation implants (1970-1982) had thin shells, thin gel and a round shape. Third generation implants (from 1982 onwards) had thicker shells, thicker gel and a round shape. Fourth generation implants (from 1986 onwards) have features similar to the third generation, except that they had textured surface. They are available in round as well as anatomic shape. Fifth generation implants (from 1993 onwards) have enhanced cohesive silicone gel and textured silicone surface. They are available in anatomic and round shapes.[5]

### Saline-filled implants

The inflatable saline filled implant was first reported by Arion,[6] in France, in 1965. The main advantage of using an inflatable implant was that it was possible to insert the implant through a small incision. The risk of the gel bleed was also completely eliminated. This also lessened capsular contracture rates. The problems specific to inflatable implants include deflation, visible surface wrinkles and knuckle like feel in volumetrically under-filled devices. If the device is over inflated, it may feel like a firm ball. Because of the weight of the implant, it may cause more tissue thinning, with downward displacement of the implant over time.

### Double-Lumen implants

The Double-Lumen implant was introduced by Hartley,[7] to prevent capsular contracture. It has inner silicone gel-filled lumen, surrounded by an outer saline inflatable shell. Reverse double-lumen implants are also available, in which the outer silicone gel-filled shell surrounds an inner inflatable shell.

### Textured surface implants

These were primarily introduced to maintain its position, but the clinical use seemed to show a decreased incidence of capsular contracture.[8]

### Anatomic and round shaped implants

The Cronin and Gerow silicone gel implant had a teardrop shape. Since there was a problem with capsular contracture, manufacturers began to design round, smooth-surfaced low-profile implants, which would move within their surgical pockets. The selection would be made according to the patient's need.

There are many varieties of silicone gel implants and saline implants available in varying degree of projection, height and shapes now.

## RELEVANT ANATOMY

The roughly circular body of the female breast rests on a bed that extends transversely from the lateral border of the sternum to the midaxillary line and vertically from the 2^nd^ through 6^th^ ribs. Two thirds of the bed of the breast are formed by the pectoral fascia overlying the pectoralis major, the remaining by the fascia covering the serratus anterior. Between the breast and the pectoral fascia is a loose connective tissue plane or potential space called the retro mammary space (bursa). This plane, containing a small amount of fat, allows the breast some degree of movement on the pectoral fascia. A small part of the mammary gland may extend along the inferolateral edge of the pectoralis major toward the axilla (armpit), forming an axillary process or tail (of Spence).

The arterial supply of the breast derives from the

- Medial mammary branches of perforating branches and anterior intercostal branches of the internal thoracic artery, originating from the subclavian artery.
- Lateral thoracic and thoracoacromial arteries, branches of the axillary artery.
- Posterior intercostal arteries, branches of the thoracic aorta in the 2^nd^, 3^rd^, and 4^th^ intercostal spaces.

The venous drainage of the breast is mainly to the axillary vein, but there is some drainage to the internal thoracic vein

## NERVES OF THE BREAST

The nerves of the breast derive from anterior and lateral cutaneous branches of the 4th to 6th intercostal nerves. The anterior primary rami of T1 to T11 are called intercostal nerves because they run within the intercostal spaces. Rami communicantes connect each anterior ramus to a sympathetic trunk. The branches of the intercostal nerves pass through the deep fascia covering the pectoralis major to reach the skin, including the breast in the subcutaneous tissue overlying this muscle. The branches of the intercostal nerves thus convey sensory fibres to the skin of the breast and sympathetic fibres to the blood vessels in the breasts and smooth muscle in the overlying skin and nipple.

### Indication for augmentation mammoplasty

- Hypomastia
- Tubular breast
- Breast reconstruction
- Cosmetic correction

### Preoperative investigations

- Complete haemogram, blood sugar, urea and creatinine
- Bleeding profile
- Chest X-ray
- Mammogram

### Assessment

A thorough physical assessment should be done, prior to the operation. The bone and muscle structural foundation of each breast must be assessed. Note the shape of the thorax. Also note whether the patient is “long” or “short” chested. Key measurements include suprasternal notch to nipple distance, nipple to inframammary fold distance, base width or diameter, and breast height. Characterize the elasticity of the skin by noting evidence of poor compliance such as stretch marks or thin nonelastic dermis.

It is also important to characterize the breast parenchyma itself. The amount, quality, and distribution of the parenchyma may alter surgical techniques

### Incisions

Four types of incisions are employed in breast augmentation

1. Transaxillary
2. Inframammary
3. Periareolar
4. Transumblical

After implant selection, the decision regarding the type of incision to be used should be made by the patient and surgeon after the options, risks, and benefits of each have been thoroughly explained.

The inframammary incision permits complete visualisation of either the prepectoral or sub glandular pockets and allows precise placement of virtually all implants. The technique does leave a visible scar within the inframammary fold.

The periareolar incision is placed at the areola-cutaneous juncture and generally heals inconspicuously. The dissection allows easy adjustment of inframammary fold and direct access to the lower parenchyma for scoring and release, when constricted lower pole is present. The disadvantages include limited exposure of surgical field, transection of the parenchymal ducts, potentially increased risk of nipple sensitivity changes, and visible scar on the breast mound.

**Figure 1:**
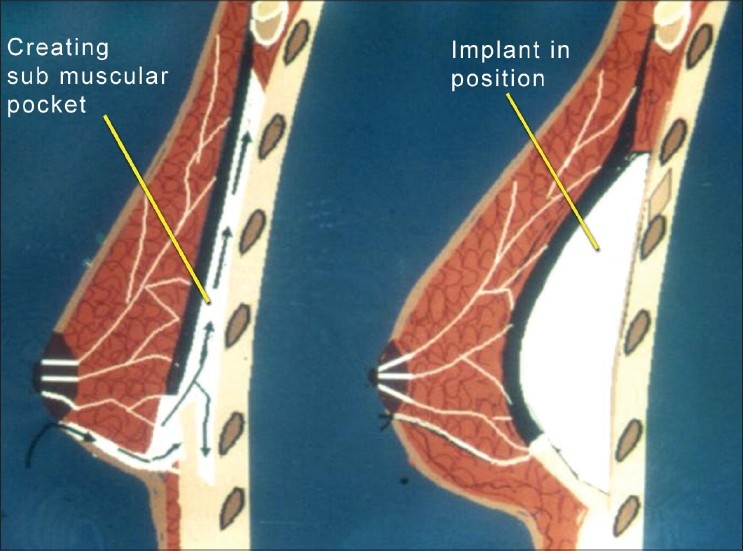
Diagram illustrating approach to placement of implant. Arrow shows dissection and development of sub muscular pocket for placement of implant

**Figure 2:**
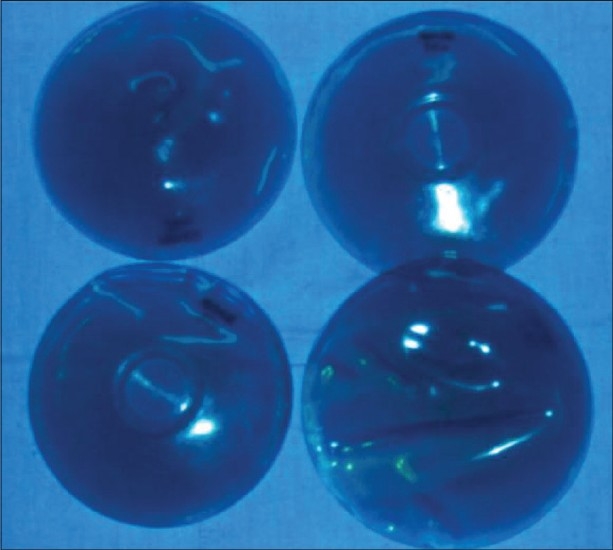
Sizing implants

The transaxillary incision can be done either bluntly or with the aid of the endoscope. This approach avoids any scarring on the breast mound. It can be used with both saline and gel filled implants in either a sub pectoral or sub glandular pocket. The problems with this approach are difficulty with parenchymal alterations and probable need for a second incision in the breast mound for secondary correction surgeries. Placing the implant in proper position may be difficult.

Transumblical breast augmentation has the great advantage of a single, well hidden, remote incision. Only saline implants can be used in this approach. Obtaining haemostasis is a problem from this remote access port.

## THE IMPLANT POCKET

### Prepectoral

Earlier, augmentation mammoplasty procedures involved limited blunt dissection in the sub glandular, prepectoral muscle plane, and typically produced pockets only slightly larger than the implants themselves. When it was realized that the forces of wound contraction during healing act to further reduce the size of the cavity, emphasis once again shifted to creating and maintaining a generous pocket. The exception is textured prostheses, which must be placed in pockets that precisely correspond to the size of the implant, so as to minimize the risk of malposition.

**Figure 3:**
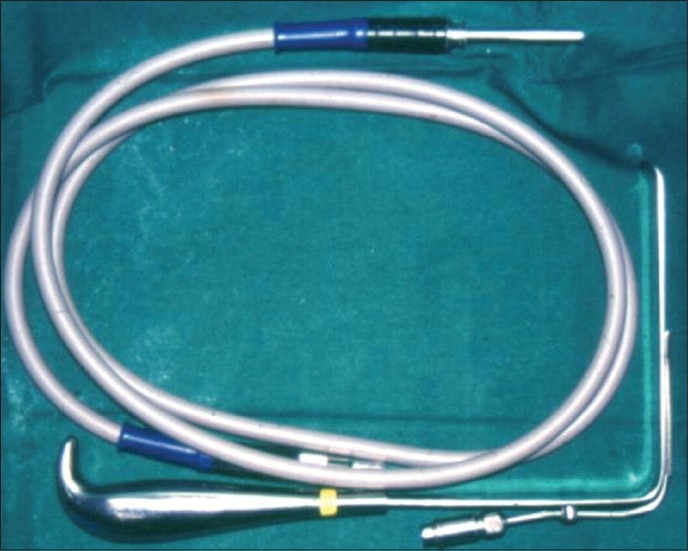
Fibre optic cable and retractor

**Figure 4:**
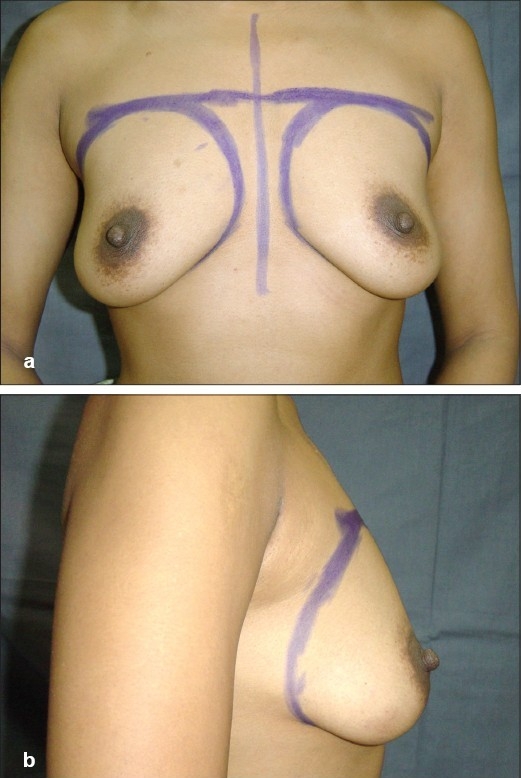
(a and b) Preoperative front and side views

**Figure 4:**
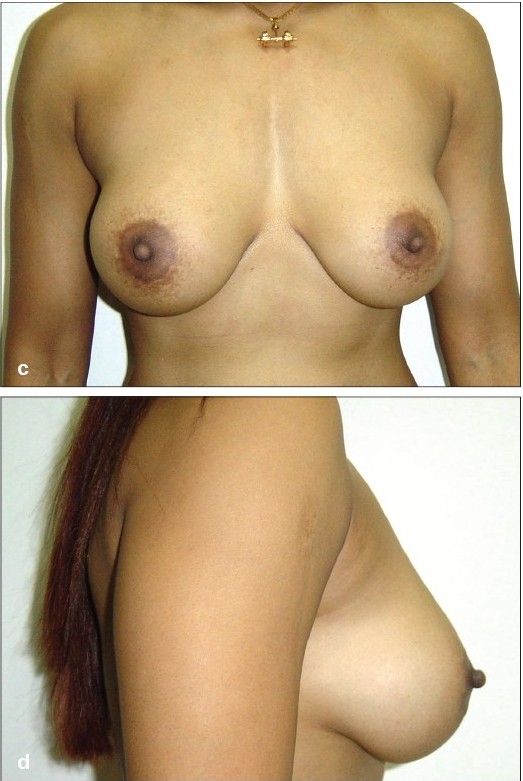
(c and d) Postoperative front and side views

Generally speaking, sub glandular implants will more effectively restore breast shape and correct breast ptosis than sub muscular implants. When considering prepectoral implantation, however, adequacy of soft-tissue cover of the implant is critical.

### Sub pectoral

In 1968, Dempsey and Latham[9] described the first augmentation mammoplasty procedure using sub pectoral prosthesis implantation. The concept of a sub muscular pocket has been adopted by many surgeons, for use in all patients.

The advantages of sub pectoral implantation are as follows:

- Lower incidence of capsular contracture
- Improved breast contour, since the edges of the implant are blunted by the muscle
- Less exposure of the prosthesis to bacterial contaminants from glandular tissue 35
- A plane of dissection that is less vascular than in retro glandular implant placement
- Maximal preservation of nipple sensation

The pectoral muscle can be divided at various levels, which was described as dual plane manoeuvres, to allow varying degrees of sub pectoral to sub glandular implant coverage.

An additional pocket more recently introduced and advocated by some is the sub pectoral fascial pocket. This thin layer of tough tissue is said by some to offer the advantage of sub glandular placement with a thicker soft tissue cover.

Alternative pocket locations include retro mammary, partial retro pectoral and total sub muscular.

**Figure 5:**
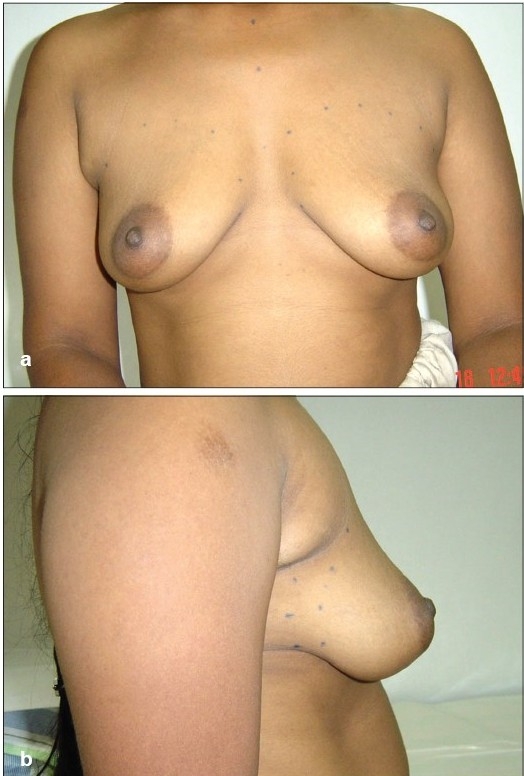
(a and b) Preoperative front and side views

**Figure 5:**
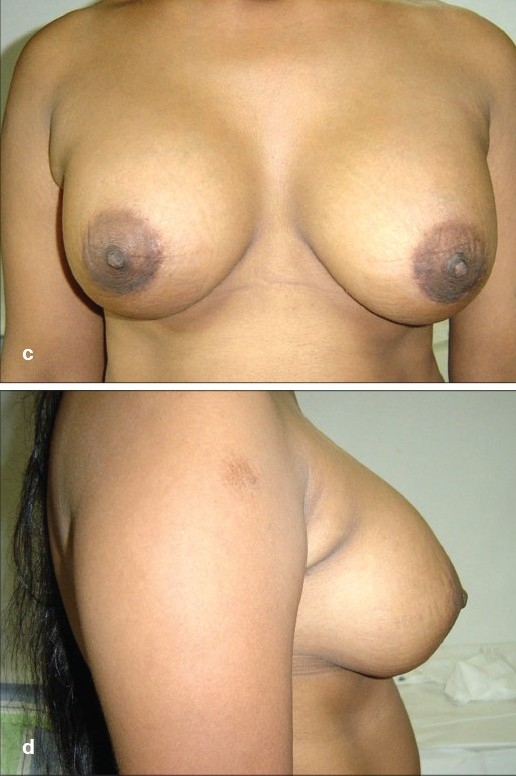
(c and d) Postoperative front and side views

### Periareolar Sub Muscular Placement

As discussed earlier, it is very important to examine the patient in order to determine the size of the chest. The pocket has also got to be marked. Keeping these dimensions in mind and also taking into consideration the elasticity of the breast skin, it is possible to arrive at some possible sizes of the implant. This, again, would depend upon the type of implant and the dimensions associated. The base diameter as well as the antero posterior projection of the implant varies not only from the low profile to the high profile or maximum high profile variety but also from company to company. Hence, the appropriate size of the implant is derived and a couple of sizes of implants on either side of the discussed volume are also kept as reserve.

The author usually performs this procedure under general anaesthesia, though it could be done under monitored anaesthesia care, with local anaesthesia.

After the markings have been done and verified, the patient is anaesthetized and positioned supine with the shoulders abducted at 90 degrees. Non talk gloves are used throughout the procedure. The areas are painted with Betadine® surgical scrub and then draped. The incision site as well as the caudal breast tissue and the peripheral markings of the pocket are infiltrated with one percent xylocaine with 1:200,000 adrenaline and about 7-10 minutes time given, in order to get a good vasoconstriction effect.

The incision is then made in the lower areola skin junction between three and nine o' clock position. This is then deepened while proceeding caudally between the breast and the subcutaneous tissue, till the pectoral fascia is reached. The breast is then lifted off the fascia for some distance cephalad. A rent is then made in the fascia and the fibres of the pectoralis major are split, so as to permit the entry of the index finger underneath the muscle. Then, with the help of a sweeping motion, the sub muscular pocket is developed. This dissection is relatively easier in the superior, medial and lateral aspects, but is usually the most difficult in the inferior pocket. Having performed most of the dissection bluntly; the rent is then widened by cutting the fibres, using a cautery, taking care to coagulate the vessels in the muscle fibres. The inferior dissection can be done better now and, if necessary, cautery can be used if blunt dissection is difficult. It may be necessary to lower the inframammary crease if the areola crease distance is less than 5 cm. This lowering will help in better centralization of the implant and the nipple areola complex. Haemostasis is secured with the help of retractors illuminated by fibre optic light and cautery. This is a very important step for ensuring that there is no haematoma or bleeding in the postoperative period, which could otherwise mar the result, causing capsule formation or sometimes causing the need to evacuate the haematoma.

Following this, it is the author's preference to use sizing implants in order to get an idea of the projection, ease of placement and the aesthetic result. Based on the sizer, the decision regarding the appropriate implant is made. With the sizer *in situ*, a similar procedure is carried out on the opposite side. This gives adequate time for the haemostasis on the first side. After removing the sizer, the pocket is checked for haemostasis. The gloves are then changed. The definitive implant is then taken and, under strict aseptic precautions, it is introduced into the pocket using a sterile sleeve, if it is the textured variety. This is done to help smooth entry by preventing the breast tissue from getting caught on the textured surface. Once the implant is in good position, the index finger is used to make sure that there are no areas of hold up and that the implant surface is free, without any folds or wrinkles.

The closure is then carried out using 3 ‘0” Ethilon® for the pectoral fascia and the muscle and 4‘0’ Prolene® for the breast tissue. 5‘0’ Prolene® is used for dermal subdermal closure and for skin closure. A similar procedure is then carried out on the opposite side. Fluffed gauze is then placed on the breast and a firm dressing is done. The patient is started on liquids after four hours and also put on pain relief medications. Antibiotics are continued for four days. She is advised restricted arm movements for about two weeks and started on Vitamin E 400 mg daily, from the 10^th^ day onwards. Vitamin E has been suggested to reduce capsular hardness after augmentation mammaplasty, by virtue of its known anti-inflammatory properties.[10]

Postoperative breast massage has been advocated by many, though there are others who feel that textured implants placed sub muscularly have significantly reduced incidence of capsular contracture.

The patient is advised to have the sutures removed after six days and the edges are then supported with steristrips® for another five days. The patient is also advised to wear a bra at all times to give support

### Surgical complications

1. Haematoma
2. Seroma
3. Wound infection
4. Reduced nipple sensation
  The nipple and areola are innervated by a lateral branch of the fourth intercostal nerve, with overlapping contributions from the anterior and lateral cutaneous branches of the third through fifth intercostals nerves.[11] These nerves run parallel to the corresponding arteries of the same name. To ensure nipple sensation after surgery in this area, one must preserve the anterior branches of the fourth, fifth and sixth intercostal arteries and the accompanying nerves on the lateral aspect of the breast. These branches are more easily mobilized in the sub pectoral plane than the sub glandular, and can be displaced laterally by blunt dissection in a medial to lateral direction. As the branches pass through deep fascia into the breast glandular tissue, they become relatively fixed and are more likely to be injured during extensive electrocautery or sharp dissection of the lateral pocket.
5. Skin irritation
6. Pain
7. Implant displacement
8. Implant rupture
9. Implant rippling
10. Capsular contracture

### Capsular contracture

Classification

In 1975, Baker[12] suggested a clinical method for grading the severity of capsular firmness around a breast implant used in augmentation mammaplasty [Table 1].

**Table 1:** Baker classification of capsular firmness in augmented breasts

Grade I	No palpable capsule	The augmented breast feels as soft as an unoperated one.
Grade II	Minimal firmness	The breast is less soft and the implant can be palpated, but is not visible.
Grade III	Moderate firmness	The breast is harder, the implant can be palpated easily, and it (or distortion from it) can be seen.
Grade IV	Severe contracture	The breast is hard, tender, painful and cold. Distortion is often marked.

This classification remains the most commonly used reporting system today.

### Management of capsular contracture

The incidence of capsular contracture has come down significantly, with the use of cohesive gel and thick shell implants. The sub muscular placement is also an important factor in this direction. If a capsule does occur and if the implant has been placed sub glandular then it could be replaced with a textured implant in the sub muscular plane.

Capsulectomy is another option. Closed capsulotomy is no longer advocated, as there is a distinct possibility of implant rupture.

## CONCLUSION

Breast augmentation is an extremely gratifying aesthetic procedure. With great strides in implant manufacturing, the quality of implants has improved significantly, reducing the risk of capsule formation. Periareolar incisions have resulted in acceptable scars. Sub muscular placement of cohesive gel implants has minimized the risk of capsule formation.
